# The Properties and Functions of Saliva

**Published:** 1923-08

**Authors:** Joseph H. Kauffmann

**Affiliations:** New York, N. Y., Dental Department, Bronx Hospital and Dispensary


### THE PROPERTIES AND FUNCTIONS OF SALIVA

BY JOSEPH H. KAUFFMANN, D. D. S., NEW YORK, N. Y.
Dental Department, Bronx Hospital and Dispensary

### INTRODUCTION

Dental and medical practitioners are constantly confronted by two expressions which represent factors in both normal and abnormal states of the teeth and other tissues of the oral cavity, namely, salivary alkalinity and acidity, and it seems to the writer that much more exact knowledge concerning the relationship of these biochemical terms to the conditions which present for consideration and treatment would be of inestimable value. There are frequently discussions in which these phrases arise, and it may be wondered why we so frequently juggle those words about, when as a matter of fact they are used without sufficient understanding.[1]

Alkalinity and acidity as applied to the oral cavity are conditional terms and must always be considered in relation to other factors. For instance, the mere finding that insalivated blue litmus paper becomes red, or red becomes blue, by no means indicates an accurate or constant chemical reaction of the saliva. On the other hand, such a test is unwarranted scientifically and almost useless practically. We must bear in mind that we are not dealing with test tubes of stable compounds, but changing tissue cells, differing secretions and concretions, multiple flora of pathogenic and non-pathogenic micro-organisms, the variable status of which may be influenced by either local or systemic agencies, or both.

Acidity of body fluid, according to the present conception of physiological chemists, is that condition in which the free hydrogen ions are in sufficient concentration to cause an acid reaction.[2] Alkalinity would then denote the opposite or that chemical reaction resulting from a predominating concentration of free basic ions. These determinations are founded upon the theory of ionic or electrolytic disassociation of molecules, the details of which it is not pertinent to presently discuss, since such explanation involves the realm of physical chemistry.[3, 4, 5]

Having thus briefly defined alkalinity and acidity in terms of physiological chemistry, the first question commanding our attention is: How should they be applied relatively to the normal oral cavity, the chief physio-chemical index of which is the saliva? The mixed salivary fluid is used as an index of oral acidity and alkalinity, although the normal secretions of the mucous glands which become a part of mixed saliva are a distinct fluid substance, a very complex and incompletely ascertained proteid.[6]

### THE GLANDS OF THE MOUTH AND THEIR PRODUCT

The salivary glands are of three types: First: Those which secrete an albuminous or serous fluid, namely, the parotid and Von Ebner's glands at the apex of the tongue. Secondly: Those which give forth a mucous or thicker fluid which includes the small but numerous glands of Blandin and Nuhn at the lingual surface of the tongue about the circumvallate papillæ, Weber's glands situated on the posterior border of the tongue, the palatine and the labial glands. Lastly: the mixed type from which emanate both kinds of fluid are the other two major glands, the sublingual and submaxillary, and some labial and some palatine. Of course in speaking generally of saliva we mean all of these secretions which are conglomerated in the mouth, as it is only for experimental purposes and with special preparations that the individual types are collected and studied, the details of which technique we need not here consider, as our discussion concerns chiefly mixed saliva from a clinical rather than a laboratory standpoint.

Normal mixed saliva is of alkaline reaction, although when the flow is scanty, or after eating, it may be slightly acid, due to an excess of carbonic acid. It has a specific gravity of 1.002 to 1.006 and is composed of about 99.5 per cent water and 0.5 per cent of solids. The daily secretion averages about three pints, and is greater during the day than at night.

The half per cent of solid matter consists of mucin, epithelial cells, micro-organisms, salivary corpuscles, ptyalin, an amylolytic ferment, albumin and inorganic salts. These salts are sodium chlorid, sodium carbonate, calcium phosphate, calcium carbonate, potassium chlorid, magnesium phosphate and potassium sulphocyanite.[6, 7]

Much discussion has arisen as to the relative importance of the last named salt, and therapeutical application in the preventive treatment of dental caries has been based on the brief knowledge thereof. It may be said that experimentally there have been only negative results in this direction.

Ordinarily saliva is a glistening, frothy and transparent fluid and when mixed with much mucin may be quite slimy. Upon resting in the mouth, by exposure to air, or upon standing, it becomes cloudy, due to the precipitation of calcium carbonate, the carbonic acid which previously held it in solution as a bicarbonate, escaping.

Let us next briefly discuss the functions of an alkaline saliva in order to more fully comprehend its action as an inhibitor of oral and dental disease. These are both physical and chemical.

### PHYSICAL OR MECHANICAL FUNCTIONS OF SALIVA

First. The chief action of the saliva in digestion is to moisten and soften the food, thus mechanically assisting in its mastication and deglutition. By the secretion of a thin, watery or serous saliva, activated by the chorda tympani, fine foodstuffs, such as crackers, are moistened while the sympathetic nervous system propagates a thicker mucous secretion which serves as a penetrating and lubricating fluid for heavier substances, such as meat, thus aiding in its trituration.

Second. The saliva acts as a lubricant to the mucous membrane, thus helping the food bolus to glide about the mouth, and by moistening the tongue assists in its free movement.

Third. The saliva assists in ejecting undesirable or foreign bodies.

Fourth. A healthy, alkaline saliva is usually concomitant with a copious flow, and this free supply acts as a mechanical aid in prophylaxis by washing away food remnants from tissue folds and crevices, interproximal spaces, pits and cavities in the teeth, thus assisting the tooth brush in its work. It may here be noted paradoxically that we often have patients present with advanced stages of periodontoclasia with the teeth highly polished and comparatively immune to caries, while the saliva may be acidic in character. The latter is perhaps an index of general acidosis and constitutional disturbances.

Fifth. The salivary fluid, chiefly through the reflex mechanism of the major glands, acts as a physical guide in thirst, hunger and culinary sensations. When its flow is diminished as in decrease in volume arising from lack of sufficient water in the blood or other tissues (although several factors may influence this phenomenon), the oral tissues become dry and the sensation of thirst is encountered. On the other hand, if we are hungry and hear of, taste, smell, or see food, its flow is increased in order to assist in the anticipated physiological fermentation of foodstuffs, and even if we are not in the aptitude for culinary dispersions the sight, hearing, smelling, or tasting of food may increase our desire for eating through a corresponding increase in salivary flow.[8]

Sixth. It is harmonious to the tissue cells of the mucous membrane and to their healthful activity, due to its moistening, cleansing and refreshing properties; and it possesses some unknown quantity which assists in giving the normal epithelial lining of the mouth an amazingly high degree of resistance.[9]

Seventh. The saliva has a solvent action upon food substances which develops their sapidity or ability to stimulate the taste glands and sensory nerves of the tongue. Through a reflex nervous mechanism, there is, as a result, an increase in salivary flow.[13, 14]

### BIOCHEMICAL FUNCTIONS OF THE SALIVA

First. An alkaline saliva is conducive to the maximum activity of the amylolytic ferment ptyalin, which is usually the only digestive juice present in the mouth. This forms dextrose and maltose which are simple sugars, from starch and complex sugars. A slight content of maltase, another ferment possibly present, may convert maltose to maltase. Catalase and oxydase have also been mentioned as minor ferments in the saliva.[6]

Second. An alkaline saliva may dissolve such minerals as are soluble in an alkaline medium.

Third. On fats it may produce a feeble emulsion.[10]

Fourth. On proteins its alkaline reaction helps to soften the muscle bundles of meat.[10]

Fifth. An alkaline saliva assists in a diminution of zymogenic microorganisms, many of which are acidophilic in nature. It thus helps to diminish the initial agent of dental caries, which in the form of some type of lactic acid (but not lactic acid itself, as this has never been actually isolated in pure form), destroys the inorganic content of enamel or cementum, as the case may be.

Sixth. Salivary alkalinity tends to prevent the accumulation of saprophytic products, arising from the decomposition of carbohydrate food remnants by this class of microorganisms and thus saliva acts as a direct inhibitor of dental decay.

Seventh. An alkaline saliva produces a deposit of salivary calculus upon the teeth because of the precipitation of calcium salts, which are normally held in solution by carbonic acid as previously stated. When these salts are precipitated upon the roughened or etched enamel especially at the gingival margins, the accumulation seems to act as retarding agent to dental caries, if kept in minimum normal amount. This form of so-called tartar may then be present without any pathological involvement.

Eighth. In an alkaline saliva mucin secreted by salivary and mucous glands is soluble, so that its cumulative action upon the soft tissues and teeth is not alone prevented, but its tenacious characteristic of holding microorganisms of decay is partly at least dissipated.

Ninth. The saliva serves frequently but not necessarily as an index of blood alkalinity, neutrality or acidity. It should be observed that acidity of the blood may become incapable of neutralization by the bases present in it. When a condition of acidosis occurs, as seen, for instance, in the uric acid diathesis, or in the various disorders of metabolism, acidity may be absolute or relative. That is, if the acid content is increased numerically over the normal it is absolute, whereas if the bases are decreased from the normal it is relative, because the actual acid content remains constant.[11] According to some writers the normal blood reaction may be neutral.[12] This then would correspondingly influence the salivary reaction.

### CONCLUSION

Having elucidated the foregoing functions of saliva, it must be said that some of them are unsettled and yet vague. It is the writer's opinion that the saliva is a potentially indicative fluid holding a horde of unsolved possibilities which will be more fully appreciated when developed by further study. Is it too much to say that we may some day be able to make, for instance, a practical clinical or laboratory salivary test for syphilis or tuberculosis? Some such tests have already been devised but owing to incompleteness have not been adopted. Perhaps also some important metabolic processes and their abnormalities may be connoted by the saliva.

The point is that this fluid is primarily a derivative of the blood, and may therefore hold properties normal and abnormal that are present in its mother fluid. It must not be looked upon as merely saliva.

It can be seen then, that normal saliva is usually, although not necessarily, alkaline, and conversely acidity would denote usually, but not always, a pathological state or at least a deviation from the normal. As a means of having a standard at hand, we should bear this in mind when we are treating conditions under which saliva may become acid in reaction, absolutely or relatively, particularly where such variation may be corrected by the dentist.

The latter subject is now under consideration, and in a later communication the writer hopes to discuss this with a view to citing practical applications of the therapy involved and conclusions derived therefrom.—*Dental Cosmos.*

---

BIBLIOGRAPHY

[1] PRINZ, H.: "Efficiency of Oral Preparations." *Journal of the Allied Dental Societies*, 1916, x, p. 210.

[2] Notes and Comments: "Acidosis." *Journal American Medical Association*, 1916, lxvi, p. 376.

[3] PRINZ, H.: "Electro-sterilization of Root-canals." *Dental Cosmos*, 1917, lix, p. 373.

[4] SIMON, W., AND D. BASE: "Chemistry." 1909, Lea and Febiger, Philadelphia.

[5] FETTE, G. T.: "Ionic Medication." *Journal National Dental* Association, 1917, iv, p. 255.

[6] FOSTER, M.: "Physiology." 1904, Macmillan, New York.

[7] HALLIBURTON, W. D.: "Physiology." 1908, Blakiston, Philadelphia.

[8] CRILE, G.: "Man an Adaptive Mechanism." 1916, Macmillan, New York.

[9] BLACK, G. V.: "Special Dental Pathology." 1915, Medico-Dental Publishing Co., Chicago.

[10] HAMMARSTEN, O.: "Physiological Chemistry." Sixth Edition, translated by Mandel. John Wiley, New York.

[11] HEMMETER, J. C.: "Calcium Metabolism in Acidosis." *Dental Cosmos*, 1914, lvi, p. 1147.

[12] DELAFIELD, F., AND T. PRUDDEN: "Pathology." 1911, Wood, New York.

[13] KIRK, E. C.: Personal communication. 1923.

[14] PICKERILL, H. P.: "The Prevention of Dental Caries and Oral Sepsis." 1914, S. S. White, Philadelphia.

